# Dietary quercetagetin attenuates H_2_O_2_-induced oxidative damage and preserves meat quality in broilers by modulating redox status and Nrf2/ferroptosis signaling pathway

**DOI:** 10.3389/fvets.2025.1713728

**Published:** 2025-12-04

**Authors:** Wenyue Hu, Huiqing Liang, Pengyu Zhao, Sijia Feng, Yuhan Li, Fengyang Wu, Shuaijuan Han

**Affiliations:** 1 College of Animal Science and Technology, Hebei Agricultural University, Baoding, China; 2 Chenguang Biotechnology Group Co., Ltd., Handan, China; 3 Hebei Province Plant Source Animal Health Products Technology Innovation Center, Handan, China

**Keywords:** broiler, meat quality, oxidative stress, quercetagetin, Nrf2/ferroptosis

## Abstract

In modern poultry production, oxidative stress has emerged as a pivotal factor compromising the health status and overall performance of broiler. The aim of this study was to investigate the effects of dietary quercetagetin (QG) supplementation on hydrogen peroxide (H_2_O_2_)-induced oxidative damage in breast muscle of broilers, focusing on growth performance, meat quality, and antioxidant function, and elucidating the underlying mechanisms. Two hundred and forty one-day-old Cobb broilers were randomly divided into three treatment groups: the control group, the H_2_O_2_ group and the H_2_O_2_ + QG group. The control and H_2_O_2_ groups were fed a basal diet, and the QG group was fed a basal diet supplemented with 100 mg/kg QG. The control group was intraperitoneally injected with normal saline, and the other two groups were treated with the same volume of 10% H_2_O_2_ solution on day 37. The experimental period was 42 days. The results showed that H_2_O_2_-induced oxidative stress increased the levels of drip loss, cooking loss, reactive oxygen species (ROS), and oxidation products in the breast muscle, and damaged the mitochondrial function. Compared with the control group, the mRNA expressions of glutathione peroxidase (*GSH-Px*), NAD(P)H quinone dehydrogenase 1 (*NQO1*), catalase (*CAT*), nuclear factor erythroid 2-related factor 2 (*Nrf2*), transferrin receptor protein 1 (*TFR1*), and ferritin heavy chain 1 (*FTH1*) in the breast muscle were decreased (*p* < 0.05). The addition of QG to the diet reduced the levels of ROS and oxidation products (*p* < 0.05). Meanwhile, the addition of QG to the diet increased the mRNA expressions of *Nrf2* and *TFR1*, showing no significant difference from those of the control group. In conclusion, H_2_O_2_-induced oxidative stress impairs breast muscle quality, mitochondrial function, and antioxidant capacity in broilers. Dietary QG alleviates oxidative stress and improves meat quality by regulating the Nrf2 signaling pathway and ferroptosis-related mechanisms. This mechanism-based finding supports QG as a safe and effective dietary additive for broiler production, providing a practical solution to enhance animal health, stabilize meat quality, and promote the sustainability of intensive poultry farming.

## Graphical abstract

Graphical Abstract 
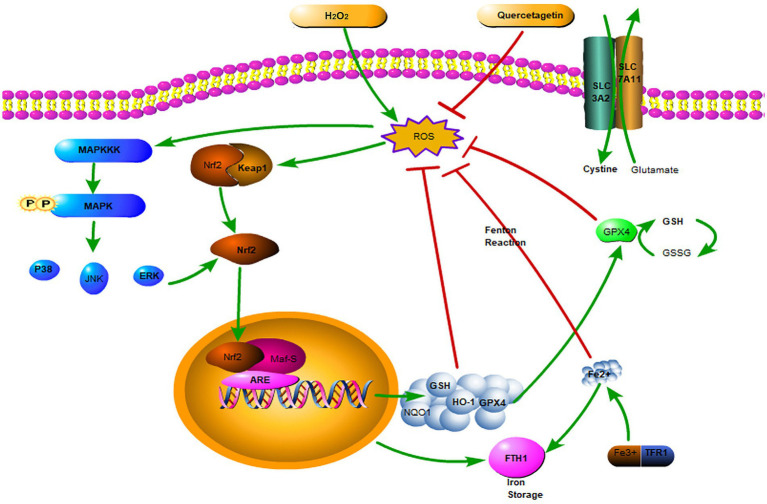

## Introduction

Nowadays, chicken meat has gained widespread popularity due to its high protein and low-fat content (1), making the preservation of meat quality a critical priority in the poultry industry. However, intensive poultry farming practices expose broilers to various stressors, including high temperatures, diseases, physiological challenges, and immune suppression (2, 3). These adverse factors induce oxidative stress in poultry (4). Accumulating evidence has demonstrated that oxidative stress generates excessive reactive oxygen species (ROS), which severely impairs broiler growth performance and meat quality (5, 6), thereby posing a significant threat to the sustainable development of the poultry industry. ROS compromise cellular integrity, increase drip loss in muscle tissues, and disrupt mitochondrial membrane potential, which in turn triggers apoptosis (7–9). This provides a rationale for using hydrogen peroxide (H_2_O_2_) as a model to induce oxidative damage in broilers. Previous studies have reported that intraperitoneal injection of H_2_O_2_ successfully induced oxidative stress and impaired meat quality (10, 11). Among various mitigation strategies, nutritional interventions aimed at scavenging excess ROS and enhancing meat quality and antioxidant capacity in broilers have attracted considerable research attention.

Quercetagetin (QG), a 3,3,4,5,6,7-hexahydroxyflavonol extracted from marigold, exhibits stronger free radical scavenging capacity and antioxidant potential compared to quercetin, attributed to its additional hydroxyl group (12, 13). Consequently, QG represents a promising natural feed additive for improving broiler health and meat quality. In our previous study, QG enhanced broiler antioxidant ability, which may through the regulation of nuclear factor E2-related factor 2 (Nrf2) signaling pathway, and reduced malondialdehyde (MDA) levels. And QG also promoted nutrient digestion by enhancing gut structure and morphology (14, 15). Furthermore, QG has been reported to alleviate zearalenone-induced liver oxidative damage in rabbits via the Kelch-like ECH-associated protein 1 (Keap1)-Nrf2-antioxidant response element (ARE) signaling pathway (16). The efficacy and potential mechanisms of QG in alleviating oxidative stress-induced muscle damage in broilers have not been reported. Especially QG’s potential role in modulating Nrf2, ferroptosis or the signaling pathway of mitogen-activated protein kinase (MAPK), which are critical in oxidative damage and cellular stress responses.

Ferroptosis, characterized by mitochondrial shrinkage, membrane rupture, reduction in internal cristae and lipid peroxidation, has been associated with oxidative stress through its dependence on ROS and reduced glutathione peroxidase 4 (GPX4) activity (17, 18). The MAPK signaling pathway is mainly used to transact extracellular stimulus signals into cells and nuclei, and causes a series of physiological and biochemical reactions. The MAPK pathway, which encompasses c-Jun N-terminal kinase (JNK), extracellular regulated protein kinases (ERK), and p38, is of great significance in modulating cellular responses to ROS-induced stress and apoptosis (19). Despite increasing evidence supporting the involvement of ferroptosis and MAPK in oxidative stress, the mechanisms by which dietary QG modulates these pathways in broilers remain poorly understood. Besides, the regulatory effects of dietary quercetagetin on ferroptosis via Nrf2 signaling in broiler muscle have not yet been investigated.

Therefore, in this study, oxidative stress was induced in broilers via H_2_O_2_ injection to evaluate whether dietary QG supplementation could mitigate H_2_O_2_-induced oxidative damage and preserve meat quality through modulating apoptosis, ferroptosis, Nrf2 or MAPK signaling pathways. These findings aim to develop a novel dietary strategy for sustainable poultry production under oxidative stress conditions.

## Materials and methods

### Animals and treatment

Two hundred and forty Cobb broilers at 1 day of age were procured from Hebei Jiuxing Agriculture & Husbandry Co. Ltd. (Baoding, China). All chickens were randomly allocated into three groups. In each group, there are eight replicate cages and 10 birds were placed in each cage. The birds in the control group were provided with a basal diet and an intraperitoneal injection of normal saline (1.0 mL/kg of BW). In the H_2_O_2_ group, birds were administered a basal diet and injected with the same volume of 10% H_2_O_2_ (2.96 mmol/kg BW). In the H_2_O_2_ + QG group, birds were provided a basal diet supplemented with 100 mg/kg QG and injected with an equal volume of H_2_O_2_. QG (purity exceeding 80%) was obtained from Chenguang Biotechnology Group Co. Ltd. (Handan, China). The formal trial lasted for 42 days, and the injection was performed on day 37. The basal diets for the starter phase (1–21 days) and grower phase (22–42 days) were formulated based on the Cobb 500 broiler, as described in Broiler Performance & Nutrition Supplement (20). The detailed information on the composition and nutrient levels of the basal diet is shown in Table 1. During the experiment, birds were reared and managed according to the description in the Broiler Performance & Nutrition Supplement. The animal experiments were carried out in strict compliance with the ARRIVE guidelines, and the experimental protocols received approval from the Committee for Animal Care and Use at Hebei Agriculture University (Baoding, China, protocol number: 2023142).

**Table 1:** Composition and nutrient levels of the basal diet (as-fed basis, %).

Items	Trial period	
	Starter phase (1–21 d)	Grower phase (22–42 d)
Ingredients		
Corn	53.87	62.03
Soybean meal	39.90	31.70
Soybean oil	2.20	2.80
Dicalcium phosphate	1.74	1.39
Limestone	0.98	0.89
Sodium chloride	0.30	0.30
*L*-Lysine hydrochloride	0.25	0.21
*DL*-Methionine	0.16	0.15
*L*-Threonine	0.10	0.03
Premix^1^	0.50	0.50
Total	100.00	100.00
Nutrient levels^2^		
Dry matter	90.26	90.06
Metabolizable energy (MJ∙kg^−1^)	12.45	12.97
Crude protein	21.75	19.23
Calcium	0.89	0.76
Total phosphorus	0.75	0.66
Non-phytate phosphorus	0.59	0.53
Digestible amino acid		
Lysine	1.22	1.02
Methionine	0.46	0.42
Threonine	0.83	0.66
Tryptophan	0.24	0.20
Valine	0.95	0.82

^1^The premix provided the following per kilogram of diets (as-fed basis): Vitamin A, 10,000 IU; Vitamin D3, 5,000 IU; Vitamin E, 80 IU; Vitamin K3, 3 mg; Vitamin B1, 3 mg; Vitamin B2, 9 mg; Vitamin B6, 4 mg; Vitamin B12, 20 μg; Pantothenic acid, 15 mg; Nicotinic acid, 60 mg; Folic acid, 2 mg; Biotin, 0.15 mg; Choline chloride, 1,000 mg; Copper, 15 mg; Iron, 40 mg; Zinc, 100 mg; Manganese, 100 mg; Selenium, 0.35 mg; Iodine, 1 mg.

^2^ME and digestible amino acid were calculated values, while the remaining nutrient levels were determined values.

### Sample collection and preparation

Following a 12 h fasting period, feed intake and BW of broilers were recorded on the 37th and the 42th days to determine the growth performance. One bird per cage was selected based on proximity to the average BW. Then these birds were euthanized and immediately slaughter thereafter for breast muscle collection.

### Meat quality measurement

The determination of the quality of breast muscle was carried out according to the research methods reported in previous studies (21). Briefly, the pH (pH_45 min_ and pH_24 h_) of breast muscle was determined at three distinct points using an electronic pH meter (Testo-205) and averaged. The meat color, represented by L* (lightness), b* (yellowness) and a* (redness), was determined using a colorimeter (Iwave W R-18). Drip loss was measured by weighing fresh breast meat in bags and suspending them at 4 °C for 24 h. The breast muscle was blotted dry and weighed again. Cooking loss was measured by weighing the breast muscle in bags, cooking them in water for 15 min, and weighing them again after cooling. The cooked samples were evaluated with a digital meat tenderness meter (C-LM3), and shear force values were recorded.

### Meat composition determination

The breast muscle samples were lyophilized for further analysis. The contents of moisture (method 935.13), crude protein (method 976.05), ether extract (method 942.05), and ash (method 2003.05) in breast muscle were determined according to the method in Association of Official Analytical Chemists (AOAC) (22). The final data were expressed as fresh weight basis.

### Free amino acids analysis

Sample pretreatment for the detection of free amino acids in breast muscle was performed as described by Oh et al. (23) and Dai et al. (24). Samples were analyzed using a high performance liquid chromatography (HPLC) (Thermo Fisher Scientific, United States) instrument. The methods for sample determination and analysis were performed as described by Boz et al. (25).

### Measurement of oxidative parameters and ROS

The breast muscle samples were homogenized with normal saline, and then centrifuged to obtain the supernatant for the subsequent determination of antioxidant indices (1). The total protein content of breast muscle was determined using BCA protein kit (Beijing Solarbio Science & Technology Co., Ltd., Beijing, China). The content of total antioxidant capacity (T-AOC, Catalog number: YX-200118C), total superoxide dismutase (T-SOD, Catalog number: YX-191504C), glutathione peroxidase (GSH-Px, Catalog number: YX-071908C), catalase (CAT, Catalog number: YX-030120C), malondialdehyde (MDA, Catalog number: YX-130401C), 8-hydroxy-2′-deoxyguanosine (8-OHdG, Catalog number: YX-081508C), advanced oxidation protein products (AOPPs, Catalog number: YX-011516C), protein carbonyl (Catalog number: YX-160315C), total sulfhydryl (Catalog number: YX-202019C) and ROS (Catalog number: YX-181519C) were measured. The corresponding commercial kits were purchased from the Beijing Borui Changyuan Technology Co., LTD. The assay procedure was performed in accordance with the kit instructions.

### Mitochondrial function analysis

The production levels of mitochondrial complexes I (MRCCI, Catalog number: YX-031513C), complexes III (MRCCIII), and mitochondrial O^2−^ (Catalog number: YX-150200C) and the changes of mitochondrial membrane potential in breast muscle tissue were measured by corresponding kits. The corresponding commercial kits were purchased from the same company as described above.

### Quantitative real-time PCR assay

Total RNA was extracted with Trizol reagent (TaKaRa, Japan). The purity of the total RNA was evaluated using a NanoDrop 2000 spectrophotometer (Thermo Fisher Scientific, United States). Subsequently, the RNA was subjected to reverse transcription to synthesize cDNA by means of a PrimeScript RT Master Mix kit (TaKaRa, Japan). RT-qPCR was carried out according to the 7500 HT Sequence detection system with SYBR Premix Ex Taq (TaKaRa, Japan).

The primers were synthesized by Tianjin Qingke Biotechnology Co., LTD (Tianjin, China), with sequences provided in Supplementary Table S1. The β-actin gene was served as the internal reference for normalizing the target genes. The relative mRNA expression was computed by the 2^−∆∆Ct^ method (26).

### Statistical analysis

Growth performance was measured with duplicate cages serving as the experimental units. The remaining data were examined using individual broilers as the experimental units. The comprehensive data were analyzed via the one-way ANOVA function in SPSS software (SPSS 26). The disparities between the means of each treatment were appraised using the Tukey’s HSD test within SPSS software. Statistical significance was defined as *p* < 0.05. Data were presented as means and SEM.

## Results

### Growth performance

Before H_2_O_2_ challenge, no differences were observed in broiler growth performance such as average daily gain (ADG), average daily feed intake (ADFI) and feed to gain ratio (F/G) among groups (Table 2). After H_2_O_2_ challenge, compared with the control group, the body weight gain rate of broilers in the H_2_O_2_ group was decreased by 34.62%. When compared with the H_2_O_2_ group, dietary supplementation with QG increased the body weight gain rate of broilers by 27.83%. However, neither of these differences reached statistical significance (*p* > 0.05).

**Table 2:** Influence of dietary QG on the growth performance of H_2_O_2_-challenged broilers.

Items^1^	Groups			SEM	*p*-value
	CON	H_2_O_2_	H_2_O_2_ + QG		
Before challenge (1–37 d)					
ADG, g	54.67	54.07	53.94	0.91	0.829
ADFI, g	99.91	99.85	100.88	1.10	0.749
F/G	1.83	1.85	1.87	0.04	0.701
After challenge (38–42 d)					
BW at 37 d of age, kg	2.06	2.04	2.04	0.03	0.839
BW at 42 d of age, kg	2.43	2.27	2.34	0.05	0.142
BW change rate, %	17.59	11.50	14.70	1.96	0.133

^1^Control, birds were provided with a basal diet and intraperitoneal injected with a normal saline solution at 1.0 mL/kg of BW; H_2_O_2_, birds were also provided with a basal diet and were injected intraperitoneally with 10% H_2_O_2_; H_2_O_2_ + QG, birds were provided with a basal diet supplemented with QG and received 10% H_2_O_2_ challenge.

^a,b^ Values in a row lacking identical superscripts or no superscripts display significant differences (*p* < 0.05).

### Meat quality measurement

Compared with the control group, the H_2_O_2_ group exhibited a significantly lower a* value and a significantly higher cooking loss in broiler breast muscle (*p* < 0.05, Table 3). Dietary supplementation with QG increased the a* value, reduced cooking loss to a certain extent and improved to the level of control group (*p* > 0.05). Additionally, in the H_2_O_2_ group, the L* value of broiler breast muscle showed an 8.68% increasing trend (*p* = 0.061) and the drip loss tended to increase 12.90% (*p* = 0.057) compared with the control group.

**Table 3:** Effect of dietary QG supplementation on meat quality in breast muscle of H_2_O_2_-challenged broilers.

Items^1^	Groups			SEM	*P*-value
	CON	H_2_O_2_	H_2_O_2_ + QG		
pH_45min_	6.83	6.87	7.07	0.14	0.211
pH_24h_	6.72	6.62	6.67	0.07	0.446
L*	42.38	46.06	43.19	1.48	0.061
a*	12.48^a^	10.01^b^	10.59^a,b^	0.76	0.014
b*	7.28	7.85	7.55	0.97	0.843
Drip loss, %	1.86	2.10	1.96	0.09	0.057
Cooking loss, %	24.82^b^	28.83^a^	27.50^a,b^	1.09	0.007
Shear force value, N	13.20	14.65	13.41	1.00	0.322

^1^Control, birds were provided with a basal diet and intraperitoneal injected with a normal saline solution at 1.0 mL/kg of BW; H_2_O_2_, birds were also provided with a basal diet and were injected intraperitoneally with 10% H_2_O_2_; H_2_O_2_ + QG, birds were provided with a basal diet supplemented with QG and received 10% H_2_O_2_ challenge.

^a,b^ Values in a row lacking identical superscripts display significant differences (*P* < 0.05).

### Nutritional composition of breast muscle

Among the three groups, no differences were observed in the contents of nutritional components (such as moisture, crude protein, ether extract, and crude ash) in broiler breast muscle (*p* > 0.05, Table 4).

**Table 4:** Effect of dietary QG supplementation on nutritional composition within breast muscle of H_2_O_2−_challenged broilers (fresh sample, %).

Items^1^	Groups			SEM	*P*-value
	CON	H_2_O_2_	H_2_O_2_ + QG		
Moisture	74.15	73.23	74.17	0.49	0.121
Crude protein	20.04	19.56	19.89	0.39	0.474
Ether extract	1.16	1.07	1.08	0.04	0.115
Crude ash	2.25	2.19	2.26	0.04	0.220

^1^Control, birds were provided with a basal diet and intraperitoneal injected with a normal saline solution at 1.0 mL/kg of BW; H_2_O_2_, birds were also provided with a basal diet and were injected intraperitoneally with 10% H_2_O_2_; H_2_O_2_ + QG, birds were provided with a basal diet supplemented with QG and received 10% H_2_O_2_ challenge.

^a,b^ Values in a row lacking identical superscripts or no superscripts display significant differences (*P* < 0.05).

### Amino acids profile

No disparities were observed in the levels of bitter amino acids, umami amino acids and sweet amino acids (Table 5) of breast muscle across the three groups (*p* > 0.05).

**Table 5:** Effect of dietary QG supplementation on free amino acids within breast muscle of H_2_O_2_-challenged broilers (‰).

Items^1^	Groups			SEM	*P*-value
	CON	H_2_O_2_	H_2_O_2_ + QG		
Val	0.24	0.24	0.24	0.07	1.000
Leu	0.28	0.30	0.29	0.07	0.971
Ile	0.16	0.16	0.14	0.06	0.952
Arg	0.11	0.15	0.12	0.05	0.710
Met	0.05	0.07	0.06	0.03	0.870
His	0.08	0.11	0.07	0.05	0.777
Phe	0.09	0.11	0.09	0.04	0.896
Tyr	0.37	0.37	0.37	0.04	0.969
Total bitter amino acids	1.39	1.50	1.38	0.39	0.948
Asp	0.32	0.32	0.26	0.10	0.789
Glu	0.61	0.66	0.61	0.18	0.950
Lys	0.37	0.40	0.36	0.13	0.966
Total umami amino acids	1.30	1.38	1.23	0.41	0.938
Thr	0.12	0.19	0.13	0.04	0.311
Ser	0.25	0.27	0.27	0.05	0.888
Gly	0.30	0.30	0.24	0.07	0.631
Ala	0.51	0.52	0.53	0.12	0.982
Total sweet amino acids	1.18	1.28	1.17	0.27	0.911

^1^Control, birds were provided with a basal diet and intraperitoneal injected with a normal saline solution at 1.0 mL/kg of BW; H_2_O_2_, birds were also provided with a basal diet and were injected intraperitoneally with 10% H_2_O_2_; H_2_O_2_ + QG, birds were provided with a basal diet supplemented with QG and received 10% H_2_O_2_ challenge.

^a,b^ Values in a row lacking identical superscripts or no superscripts display significant differences (*P* < 0.05).

### Antioxidants activity and contents of oxidative products

Compared with the control group, H_2_O_2_-induced oxidative stress increased the levels of MDA, 8-OHdG, AOPPs and Sulfhydryl in breast (*p* < 0.05, Table 6), and decreased the activities of SOD, GSH-Px, T-AOC and CAT (*p* < 0.05). Compared with the H_2_O_2_ group, broilers in the H_2_O_2_ + QG group enhanced SOD, T-AOC and CAT activities, reduced levels of MDA, 8-OHdG and AOPPs (*p* < 0.05).

**Table 6:** Effect of dietary QG supplementation on antioxidant capacity within breast muscle of H_2_O_2_-challenged broilers.

Items^1^	Groups			SEM	*P*-value
	CON	H_2_O_2_	H_2_O_2_ + QG		
SOD, U/mg of protein	86.19^a^	56.95^b^	88.98^a^	5.00	<0.001
GSH-Px, U/mg of protein	59.20^a^	40.07^b^	46.19^ab^	4.91	0.006
T-AOC, U/mg of protein	3.44^a^	2.37^b^	3.17^a^	0.20	<0.001
CAT, U/mg of protein	11.17^a^	7.49^b^	10.48^a^	0.83	0.002
MDA, nmol/mg of protein	0.28^c^	0.49^a^	0.37^b^	0.03	<0.001
8-OHdG, ng/mg of protein	2.49^b^	5.09^a^	3.47^b^	0.39	<0.001
AOPPs, p mol/mg of protein	4.20^c^	7.64^a^	5.28^b^	0.37	<0.001
Canbonyl, μmol/mg of protein	12.98^a^	11.73^b^	11.64^b^	0.44	0.017
Total sulfhydryl, μmol/mg of protein	4.93^b^	5.61^a^	5.71^a^	0.13	<0.001

^1^Control, birds were provided with a basal diet and intraperitoneal injected with a normal saline solution at 1.0 mL/kg of BW; H_2_O_2_, birds were also provided with a basal diet and were injected intraperitoneally with 10% H_2_O_2_; H_2_O_2_ + QG, birds were provided with a basal diet supplemented with QG and received 10% H_2_O_2_ challenge.

^a,b^ Values in a row lacking identical superscripts display significant differences (*P* < 0.05).

### Generation of ROS

**Figure 1:**
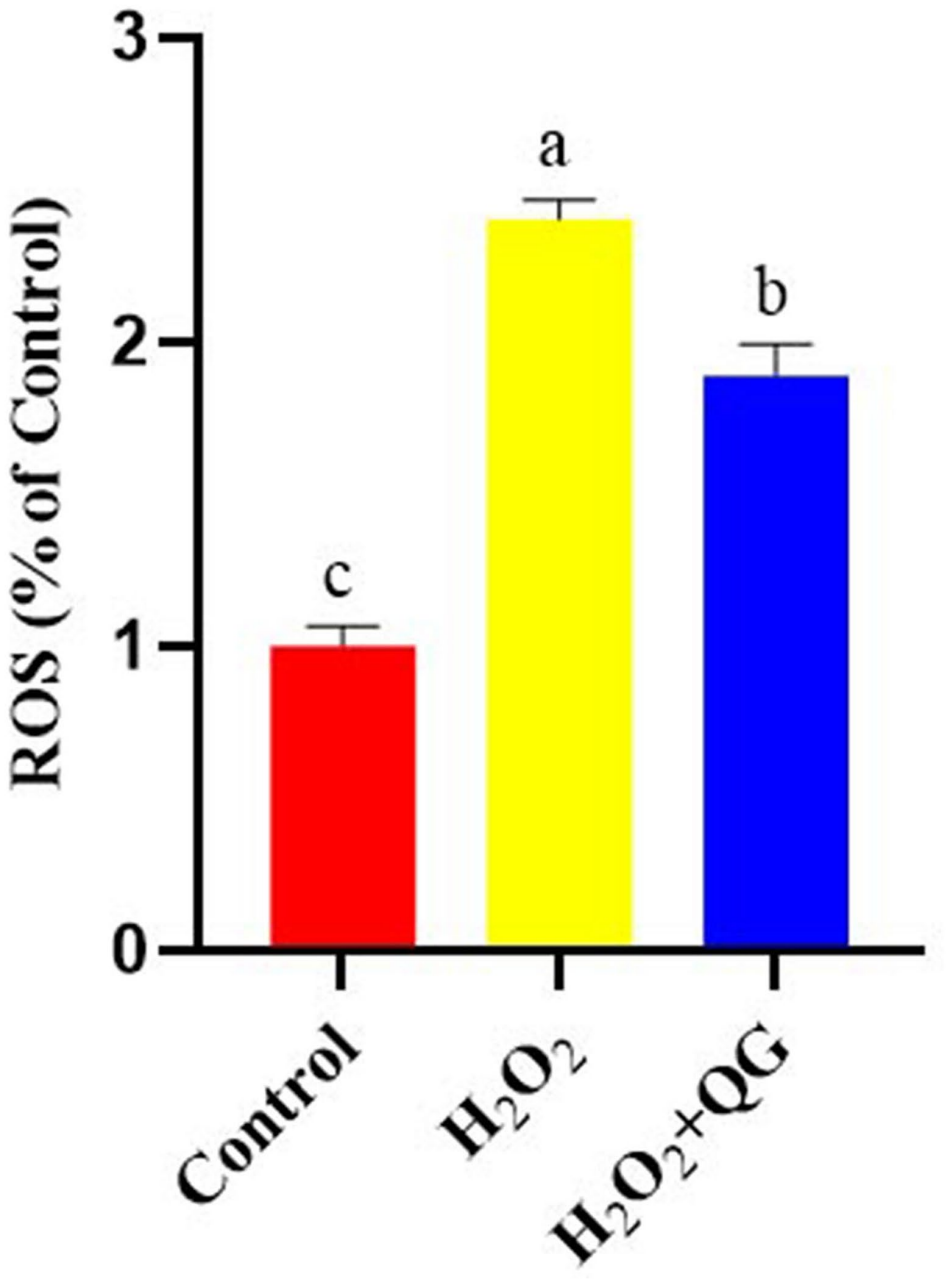
ROS production levels were measured after H_2_O_2_ and QG treatment within breast muscle.

In contrast to the control group, the H_2_O_2_ challenge led to an elevation in the ROS level by 140.28% in breast muscle (*p* < 0.05, Figure 1). The supplementation with QG mitigated the adverse effect by decreasing ROS level by 21.43% (*p* < 0.05) compared with the H_2_O_2_ group.

### Mitochondrial damage

**Figure 2:**
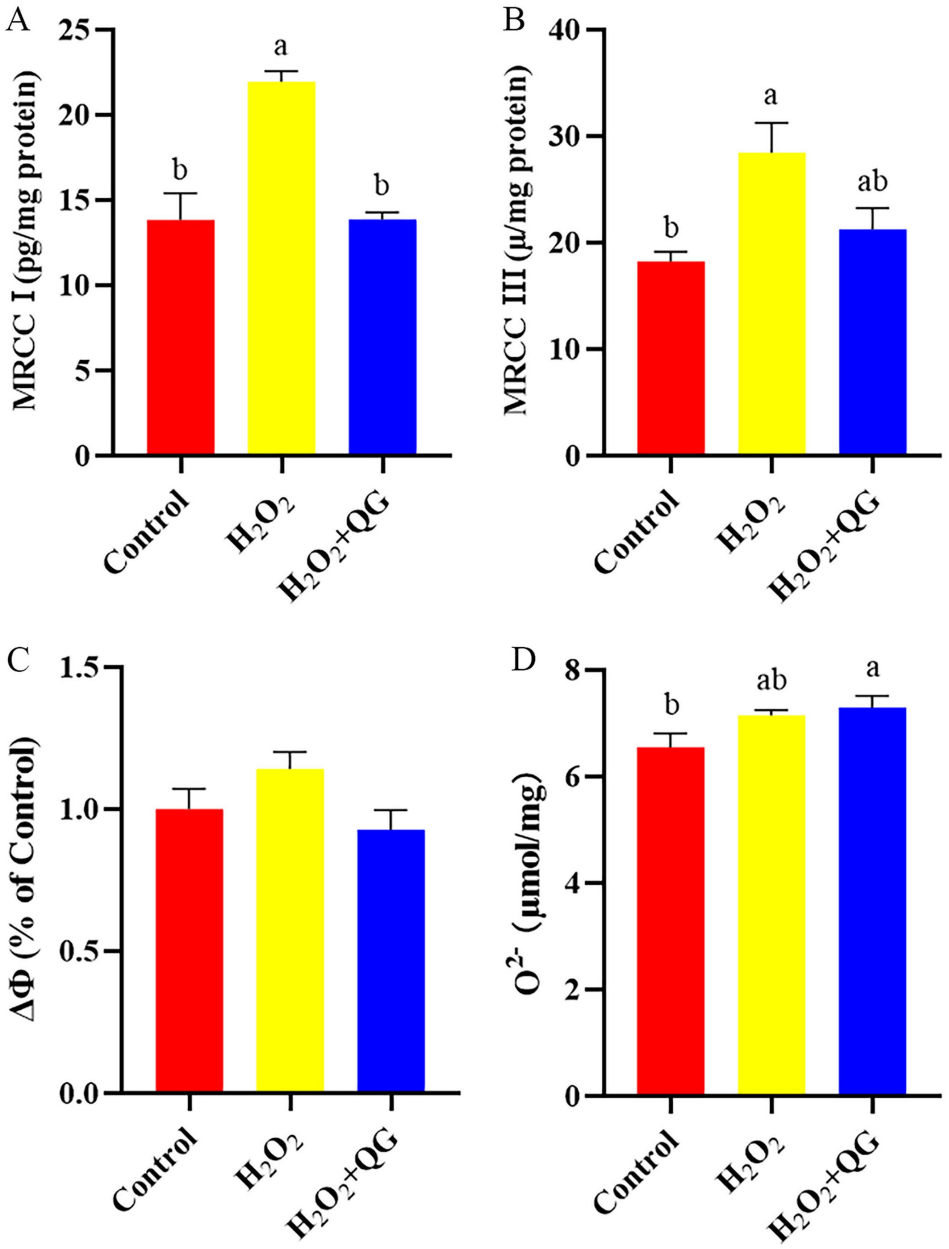
QG alleviated the symptoms of H_2_O_2_-induced mitochondrial damage in broiler breast muscle. **(A)** Level of MRCC I production. **(B)** Level of MRCC III production. **(C)** Level of mitochondrial membrane potential. **(D)** Level of O^2−^ concentration.

Compared with the control group, H_2_O_2_-induced oxidative stress increased MRCC I and MRCC III levels by 56.73 and 55.79%, respectively (*p* < 0.05, Figure 2). The addition of QG partially mitigated the adverse effects and had no difference with that in control group. The H_2_O_2_ + QG group displayed a greater O^2−^ concentration (*p* < 0.05) than the control group. No variation was detected in mitochondrial membrane potential among all groups.

### Antioxidant enzyme mRNA expression

**Figure 3:**
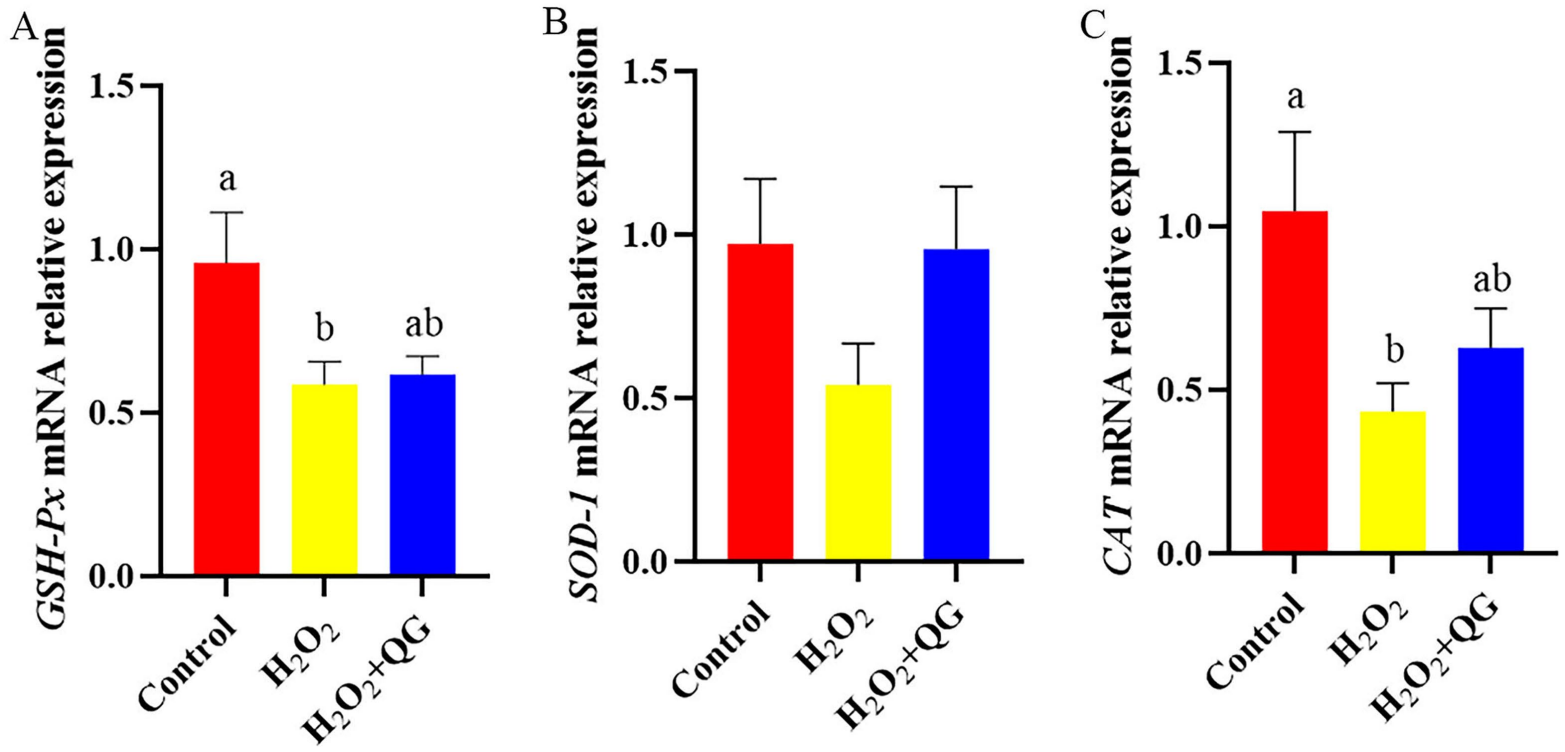
QG attenuated H₂O₂-induced dysregulation of antioxidant—related genes in broiler breast muscle. **(A)** The expression level of *GSH-Px*. **(B)** The expression level of *SOD-1*. **(C)** The expression level of *CAT*.

H_2_O_2_ challenge reduced the *GSH-Px* mRNA expression by 39.58% (*p* < 0.05, Figure 3) and reduced *CAT* mRNA expression by 58.10% (*p* < 0.05) compared with the control group. The addition of QG in the H_2_O_2_ + QG group partially mitigated this reduction in *GSH-Px* and *CAT* mRNA expression, which increased by 4.98 and 44.46%, respectively, compared to the H_2_O_2_ group, almost reaching the level of the control group (*p* > 0.05).

### Apoptosis response

**Figure 4:**
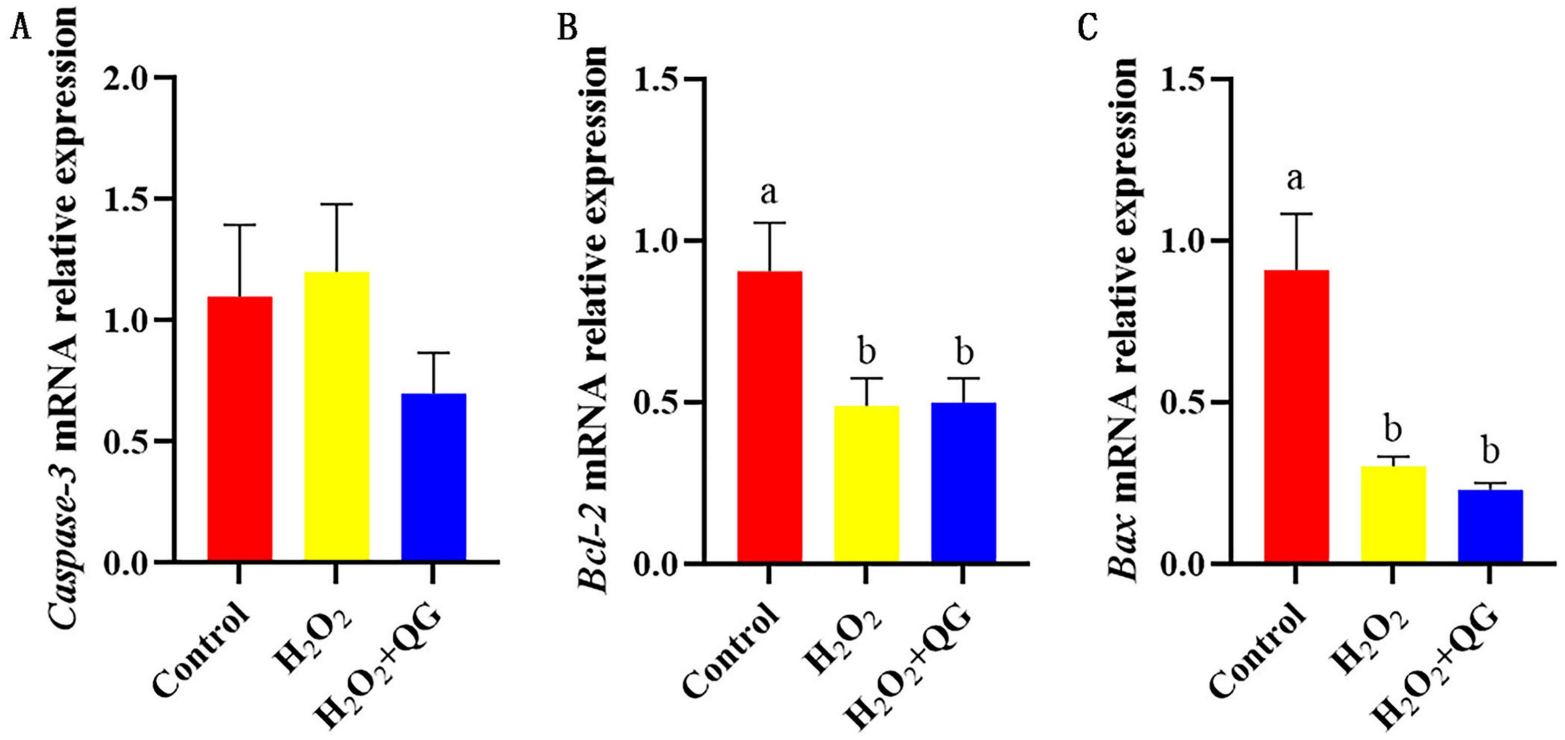
QG alleviated the dysregulation of genes related to apoptosis of cells induced by H_2_O_2_ in broiler breast muscle. **(A)** The expression level of *Caspase-3*. **(B)** The expression level of *Bcl-2*. **(C)** The expression level of *Bax*.

As shown in Figure 4, H_2_O_2_-induced oxidative stress reduced B-cell lymphoma-2 (*Bcl-2*) (by 46.15%, *p* < 0.05) and Bcl-2-associated X protein (*Bax*) (by 67.03%, *p* < 0.05) mRNA expression compared with the control group. No disparities were detected in *Caspase-3* mRNA expression among the three groups.

### Nrf2, MAPK signaling and ferroptosis

**Figure 5:**
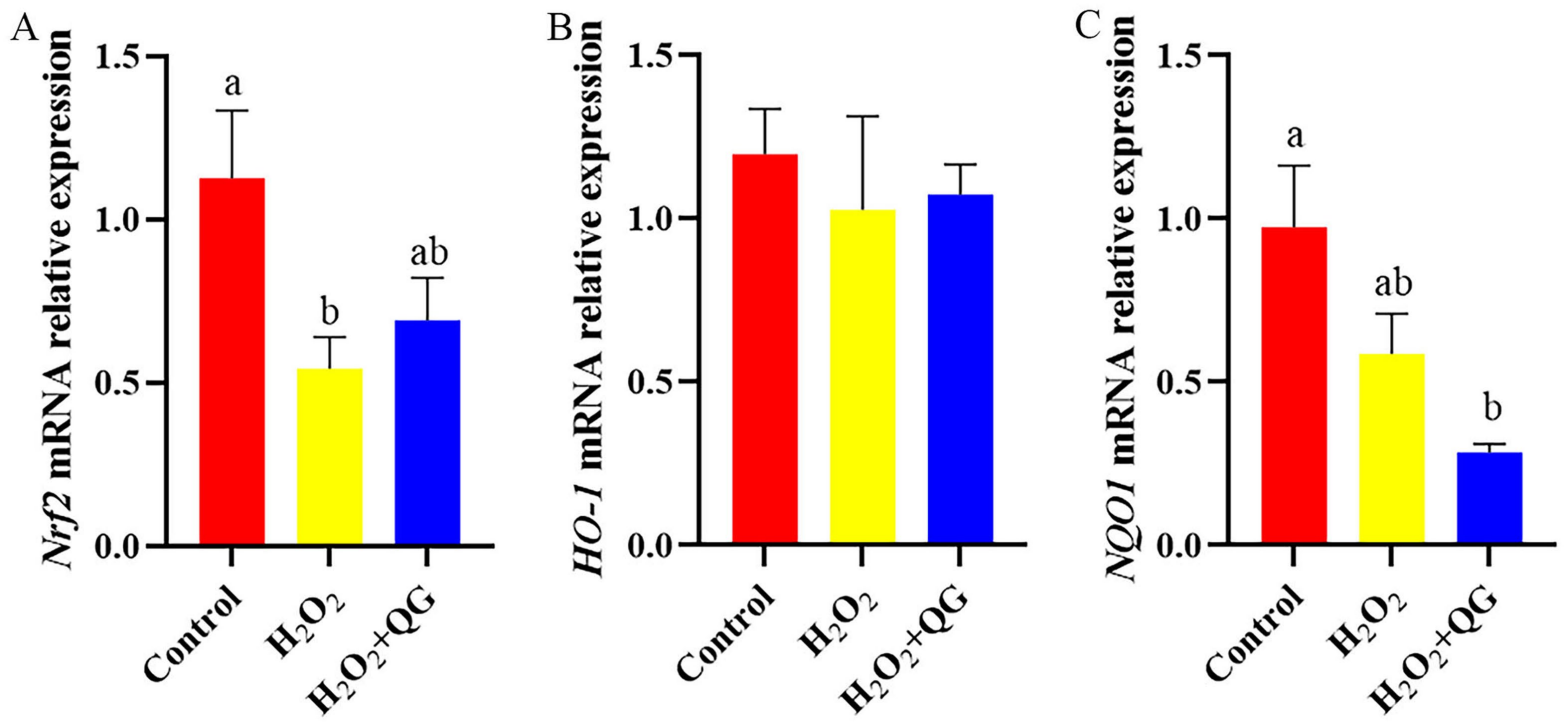
QG alleviated H_2_O_2_-induced dysregulation of Nrf2 signaling pathway related gene in broiler breast muscle. **(A)** The expression level of *Nrf2*. **(B)** The expression level of *HO-1*. **(C)** The expression level of *NQO1*.

**Figure 6:**
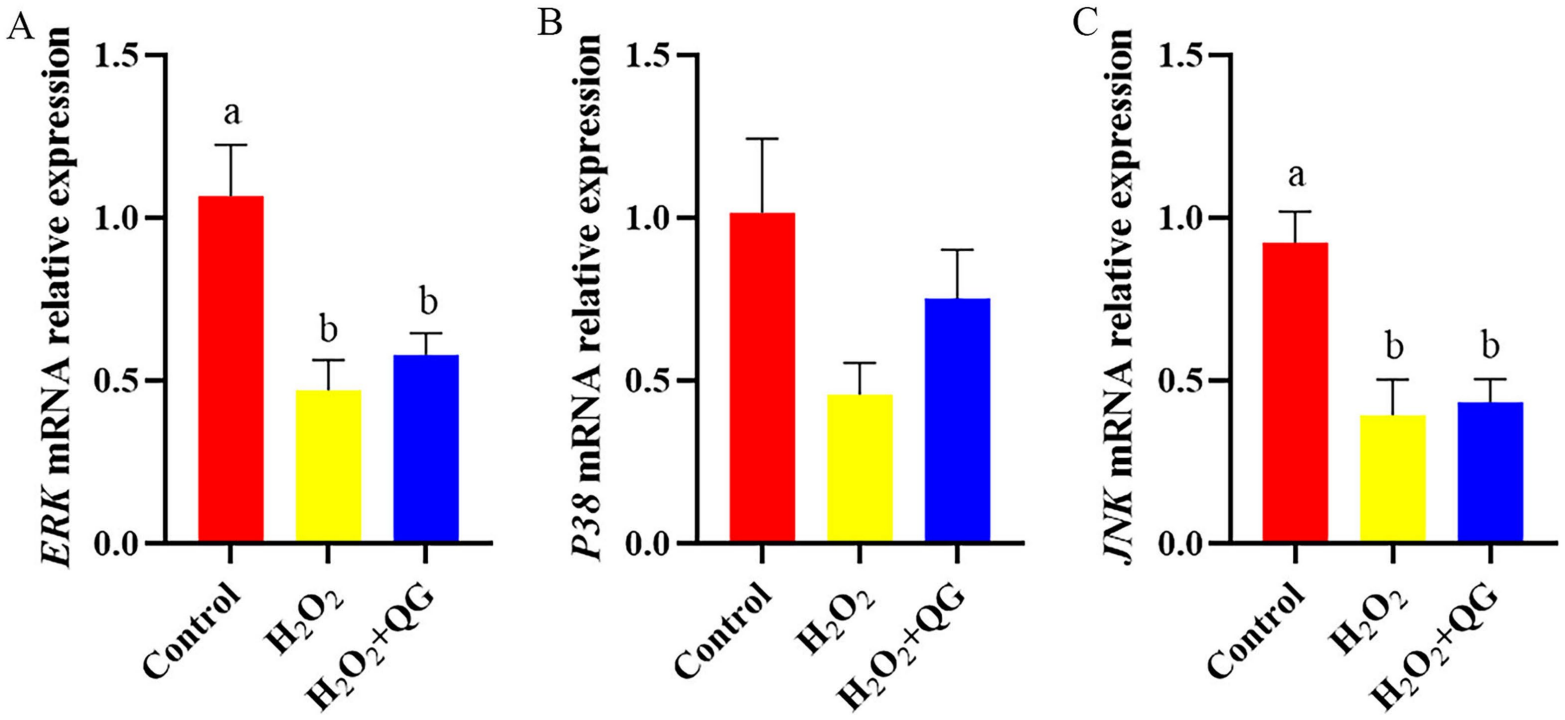
QG alleviated H_2_O_2_-induced dysregulation of MAPK signaling pathway related genes in broiler breast muscle. **(A)** The expression level of *ERK*. **(B)** The expression level of *P38*. **(C)** The expression level of *JNK*.

**Figure 7:**
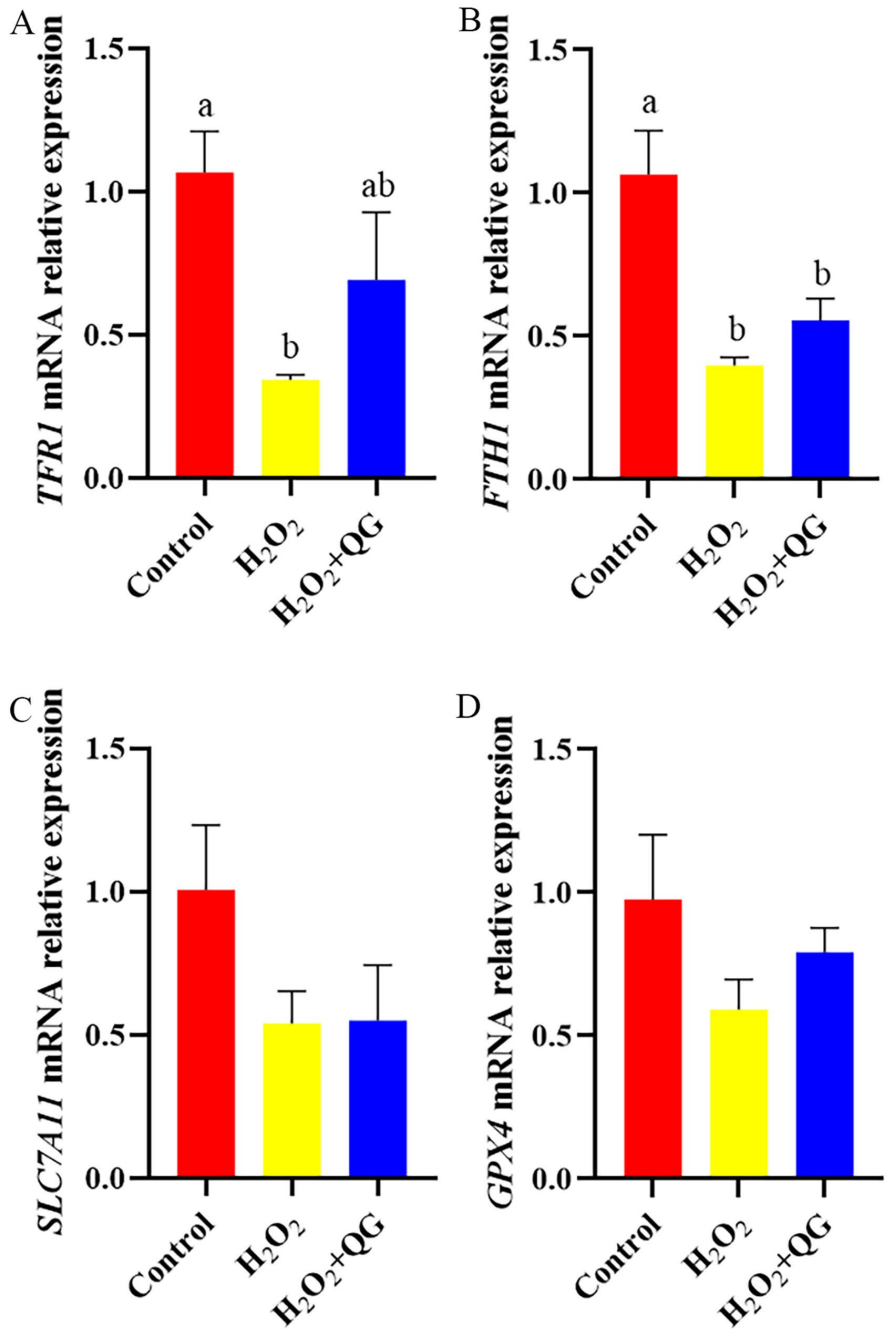
QG alleviated the dysregulation of genes involved in the ferroptosis induced by H_2_O_2_ in broiler breast muscle. **(A)** The expression level of *TFR1*. **(B)** The expression level of *FTH1*. **(C)** The expression level of *SLC7A11*. **(D)** The expression level of *GPX4*.

Compared with the control group, H_2_O_2_-induced oxidative stress reduced *Nrf2*, *ERK*, *JNK*, transferrin receptor protein 1 (*TFR1*) and ferritin heavy chain 1 (*FTH1*) mRNA expression (*p* < 0.05, Figures 5–7). The addition of QG to the diet partly counteracted the negative impacts by enhancing the mRNA expression of *Nrf2* and *TFR1*, attaining the same level as that of the control group (*p* > 0.05). There was no impact on *ERK* and *JNK* mRNA expression in breast muscle between the H_2_O_2_ and H_2_O_2_ + QG groups. No disparities were detected in *P38* mRNA expression among the three groups.

## Discussion

This study demonstrated the potential of QG to alleviate H_2_O_2_-induced oxidative stress and improve meat quality and antioxidant capacity in broilers. In this study, intraperitoneal injection of H_2_O_2_ was used to directly increase ROS levels, effectively creating an oxidative stress model, as established in prior research (27). Previous study had confirmed the detrimental effects of oxidative stress on broilers. For example, broilers treated with H_2_O_2_ exhibited reduced BW and FI and increased F/G (11). Similarly, other oxidative stress inducers, such as lipopolysaccharide (LPS) and diquat, had been demonstrated to have a negative impact on broilers (28–30). It was previously reported that the BW, feed intake of broilers increased linearly from days 1 to 35 with the addition of quercetin (31, 32). However, in this study, dietary QG supplementation did not improve broilers growth performance during the pre-challenge period (day 1–37). Wu et al. (16) also found that adding QG had no influence on the growth performance of rabbits. Previous study showed that H_2_O_2_ induced oxidative stress decreased the BW change rate of broiler after challenge (11). However, our results showed no significant differences in body weight changes after H_2_O_2_ induction. This discrepancy could be associated with variations in molecular properties, broiler breed, muscle type, age, or physiological conditions, such as the degree of oxidative stress.

Meat color is a crucial indicator of freshness (12, 33). Oxidative stress promotes glycolysis, leading to excessive production of lactate, which lowers muscle pH (2). Additionally, lipid oxidation in muscle tissue is promoted by ROS, which negatively affects meat color and stability. Secondary metabolites of lipid oxidation, such as MDA, accelerates myoglobin oxidation (11). Protein oxidation also alters the texture and flavor of meat, ultimately impairing meat quality. In this study, H_2_O_2_ challenge reduced the a* value and increased cooking loss, along with the tendency to increase the drip loss of breast muscle. Dietary supplementation with QG partially improved the meat color and reduced the cooking loss. This suggested that the addition of QG to the diet ameliorated the damage to meat quality caused by oxidative stress to some extent. QG had one more hydroxyl group than traditional quercetin in structure. Previous study showed that the addition of quercetin enhanced the a* value and reduced the b* value of breast in a dose-dependent fashion (12). Wang et al. (6) found that dietary supplementation with radix astragali suppressed lipid oxidative process, elevated a* value and lessened b* value at 24 h. Quercetin, as an external antioxidant, is capable of safeguarding lipids against oxidation and maintaining the stability of myoglobin content. Thus slowing down the process of color change in meat. Lipid peroxidation has the ability to impair the integrity of cell membranes and lead to an increment in drip loss. Dietary quercetin has been shown to promote the loosening of muscle fiber structure, reducing shear force and improving the tenderness of breast muscle (34, 35). Therefore, QG may also enhance the secondary structure and thermal stability of breast muscle proteins, ultimately achieving the goal of improving the tenderness and quality of the meat. In conclusion, QG effectively alleviated the decline in meat quality caused by oxidative stress, reduce cooking loss, and enhance the commercial value of chicken.

In addition, the content and composition of free amino acids are also important indicators affecting meat quality. Amino acids are essential components of proteins. It not only provides important nutritional value to muscle, but also plays an important role in improving muscle flavor and taste (36). The current research revealed that the addition of QG did not exert any influence on the flavor profile of broiler breast muscle. The precise mechanisms by which QG mediates these effects remain unclear and warrant further investigation.

Owing to the swift multiplication of muscle cells and the high degree of unsaturation of muscle lipids, the breast muscle is especially susceptible to oxidative stress (37). Our findings corroborated these observations, as H_2_O_2_ exposure increased ROS, MDA, 8-OHdG, AOPPs, and sulfhydryl contents, reduced the GSH-Px, and CAT activities. These results suggested that the ROS-induced damage undermines celluar antioxidant system, leading to lipid per-oxidation, protein oxidation, and mitochondrial dysfunction. These findings were in line with the ones obtained from previous investigations (10, 38). We found that dietary QG supplementation may alleviate oxidative damage by scavenging ROS, enhancing antioxidant enzyme activities, and alleviating mitochondrial impairment. Similarly, pterostilbene, a natural dimethylated analog of resveratrol, was shown to alleviate diquat-induced liver injury and oxidative stress in the liver of broilers (28). These findings were consistent with those of previous investigations (39, 40). The antioxidant effect of QG may be ascribed to its structural configuration possessing six hydroxyl groups, which may help to scavenge ROS, superoxide and hydroxyl radicals, as well as activate antioxidant defense system to alleviate oxidative damage (41, 42). Oxidative stress is also recognized to activate apoptosis pathway, leading to irreversible cell death (43). Our results showed that H_2_O_2_ impaired mitochondrial function. This indicated that the REDOX imbalance in mitochondria may inhibit the mitochondrial electron transport chain, and intracellular anaerobic respiration acts, leading to apoptosis (31, 44, 45).

Nrf2 represents a critical transcription factor in the cellular defense against oxidative stress. Under oxidative stress, Nrf2 disengages from Keap1, translocates to the nucleus, and then attaches to the ARE sequence. This process consequently triggers the transcription of downstream antioxidant genes (46). Additionally, the activation of Nrf2 is also modulated by upstream kinases, including those in the MAPK pathway (e.g., ERK, JNK, p38), which can phosphorylate and promote Nrf2 nuclear translocation (46). However, our study found that H_2_O_2_-induced oxidative stress led to a significant reduction in *Nrf2*, NAD(P)H quinone dehydrogenase 1 (*NQO1*), *ERK*, *JNK* mRNA expression, suggesting inhibition of both the Nrf2 and MAPK signal pathways. This may be due to differences in experimental conditions, such as H_2_O_2_ dose, exposure time, or animal tissues or models. Bao et al. (47) reported that simvastatin affected the expression of Nrf2/MAPK signaling pathway in *Gambusia affinis*, and exhibited a time- and dose-dependent relationship. These findings underscored the complexity of oxidative stress regulation, which might be affected by either pre- or post-transcriptional control mechanisms. Moreover, MAPKs with sulfhydryl group are susceptible to ROS (48). This highlights the close association with the regulation of Nrf2. Therefore, the decrease in the presentation of MAPK and Nrf2 pathway genes after H_2_O_2_ challenge may have a connection with ROS accumulation, inhibition of MAPK signaling pathway, and the complex cross-regulatory mechanism between MAPK/Nrf2. We found that dietary supplementation with QG activated the Nrf2 pathway, partially restoring the *Nrf2* mRNA expression to levels comparable to the control group. There were no discernible differences in relation to *ERK* and *JNK* gene expression. This suggested that QG may enhance cellular antioxidant defenses by activating Nrf2 signal pathway, whereas the involvement of MAPK signaling requires further investigation.

Ferroptosis is caused by disrupted iron homeostasis and enhanced lipid peroxidation. Under stress, cells often increase iron uptake while reducing iron storage, leading to an iron overload (49). Superfluous iron accretion is capable of facilitating the generation of ROS by means of the Fenton reaction, thereby inducing lipid peroxidation. ROS reacts with PUFA in cell membranes, resulting in membrane disruption, mitochondrial damage, and the commencement of ferroptosis. While some flavonoids such as quercetin have been shown to reduce ROS production through the Fenton reaction (50). Chen et al. (51) reported that LPS injection induced broiler intestinal inflammation and may through ferroptosis. Furthermore, the notion that flavonoids modulated the Nrf2/GPX4 axis to suppress ferroptosis is supported by a recent study, which demonstrated that 7,8-dihydroxyflavone (7,8-DHF) alleviated liver injury by promoting Nrf2 nuclear translocation and upregulating GPX4 expression (52). Our study found that dietary QG supplementation partly alleviated oxidative stress by regulating the Nrf2 signal pathway and ferroptosis-ralated genes (*Nrf2* and *TFR1*). This accorded with the results obtained by Chen et al. (53), who documented similar effects that pterostilbene supplementation to some extent enhanced the mRNA expression level of *TFH1* than diquat exposure, suggesting that QG may similarly mitigate H_2_O_2_-induced iron dysregulation. Intriguingly, this protective effect presents an contrast with the mechanism of action of quercetin in cancer cells, which inhibited the Nrf2 pathway to induce ferroptosis (54). This bidirectional regulation underscores the context-dependent nature of flavonoid activity. Furthermore, GSH and GPX4 are crucial components in the cellular antioxidant defense system. GSH plays the role of a reducing agent, whereas GPX4 is the primary peroxidase responsible (55). The synthesis of GSH is closely regulated by solute carrier family 7 member 11 (SLC7A11) (56). The addition of QG diminished the lipid peroxidation triggered by H_2_O_2_, yet it did not exert a marked influence on the mRNA expression level of *SLC7A11* or *GPX4*. The precise mechanisms underlying QG’s protective effects, particularly in modulating Nrf2 pathways and ferroptosis, remain unclear and warrant further investigation.

Collectively, the promising results of this study highlight the potential of QG as a dietary antioxidant for broilers under oxidative stress. However, the current findings are limited by the reliance on mRNA data. Future research should include dose–response studies under various challenging conditions and protein-level analyses to fully elucidate the underlying mechanisms and optimize practical application. Besides, longer-term trials would be valuable to assess the sustainability of QG’s effects.

## Conclusion

In conclusion, H_2_O_2_-elicited oxidative stress significantly impaired meat quality and mitochondrial function of breast muscle. Dietary supplementation with QG provided a protective efficacy by alleviating oxidative injury and lipid peroxidative effects, enhancing the antioxidant capacity and improving the meat quality of breast muscle. The protective effects of QG is likely mediated through the activation of the Nrf2 signal pathway and modulation of key ferroptosis-related genes. These findings provide a mechanistic basis for using QG as a novel dietary antioxidant and highlight its potential as a practical strategy to improve meat quality and animal health in sustainable poultry production.

## Data Availability

The raw data supporting the conclusions of this article will be made available by the authors, without undue reservation.
