# Supplementary material for: Dietary quercetagetin attenuates H2O2-induced oxidative damage and preserves meat quality in broilers by modulating redox status and Nrf2/ferroptosis signaling pathway
Source: Front Vet Sci. 2025 Dec 4;12:1713728. doi: 10.3389/fvets.2025.1713728 (PMC12713353; doi:10.3389/fvets.2025.1713728)
Supplement: Supplementary file 1 [file Table_1.DOCX]

Supplementary Material

# Supplementary Data

STable 1. Special primer sequences of target genes used in the present study for qRT-PCR

| Gene^1^ | Reference sequence | Primer sequence (5′to 3′) |
| --- | --- | --- |
| GSH-Px | NM_001277853.1 | F: GCTGTTCGCCTTCCTGAGAG  R: GTTCCAGGAGACGTCGTTGC |
| SOD-1 | NM_205064.1 | F: AGGGAGGAGTGGCAGAAGT  R: GCTAAACGAGGTCCAGCAT |
| CAT | XM_015274015.1 | F: TGCCCCTGTGGTCAAAGTG  R: GGTTCGGTTACCGTCCTGC |
| Nrf2 | XM_015289381.3 | F: CACCAAAGAAAGACCCTCCT  R: GAACTGCTCCTTCGACATCA |
| HO-1 | NM_205344.1 | F: CCGCTATTTGGGAGACCT  R: CTCAAGGGCATTCATTCG |
| NQO1 | NM_001277621.1 | F: TCTCTGACCTCTACGCCAT  R: TCTCGTAGACAAAGCACTCGG |
| Caspase-3 | XM_046915477.1 | F: ACAGCAAGCGAAGCAGTTTT  R: TCACCTCTGAAAAGGCTGGT |
| Bcl-2 | [NM_205339.3](https://www.ncbi.nlm.nih.gov/entrez/viewer.fcgi?db=nucleotide&id=2156850580) | F: GACAACGGAGGATGGGATG  R: CAGGCTCAGGATGGTCTTCA |
| Bax | [XM_040676625.2](https://www.ncbi.nlm.nih.gov/entrez/viewer.fcgi?db=nucleotide&id=2201813977) | F: CAACAGGAAGAACACGCTGA  R: TCAGTCTCGGCCCACTATCT |
| ERK | [NM_204150.2](https://www.ncbi.nlm.nih.gov/entrez/viewer.fcgi?db=nucleotide&id=2161398732) | F: ACCTCAGCAACGACCACATT  R: GAGCCAGTCCGAAGTCACAA |
| P38 | [XM_046920836.1](https://www.ncbi.nlm.nih.gov/entrez/viewer.fcgi?db=nucleotide&id=2201809540) | F: GCATCCATCTTCGTCGTCAT  R: TCATCTACAGCAACCCAGAGG |
| JNK | [XM_046926860.1](https://www.ncbi.nlm.nih.gov/entrez/viewer.fcgi?db=nucleotide&id=2201749199) | F: TGAAGCAGAAGCTCCTCCACCTC  R: CACTGCTGCATCTGTGCTGAGG |
| TFR1 | [NM_205256.2](https://www.ncbi.nlm.nih.gov/entrez/viewer.fcgi?db=nucleotide&id=758169980) | F: GTTATCGTGGACGAATCGAGC  R: ACACCTACCCTCCACCTCAA |
| FTH1 | [NM_205086.2](https://www.ncbi.nlm.nih.gov/entrez/viewer.fcgi?db=nucleotide&id=2099396446) | F: TACGCCTCCTACGTGTACCT  R: CTGCAAGAAGATGCGTCCAC |
| SLC7A11 | [XM_040670527.2](https://www.ncbi.nlm.nih.gov/entrez/viewer.fcgi?db=nucleotide&id=2201797369) | F: GACTGGTAGTTGCTGGCTTGA  R: TCCTCTGACGGGACAACTTC |
| GPX4 | [NM_204220.3](https://www.ncbi.nlm.nih.gov/entrez/viewer.fcgi?db=nucleotide&id=2099396645) | F: GGTGAGGCAGACCCGAAGA  R: CGTTTCCAGTGGGTTTATTTCA |
| β-actin | NM_205518.2 | F: CTTCCAGCCATCTTTCTT  R: ATATCCACATCACACTTCAT |

^1^ GSH-Px, glutathione peroxidase; SOD-1, superoxide dismutase 1; CAT, catalase; Nrf2, nuclear factor E2-related factor; HO-1, heme oxygenase-1; NQO-1, NAD (P)H quinone dehydrogenase 1; Caspase-3, cysteinyl aspartate specific proteinase 3; Bcl-2, B-cell lymphoma-2; Bax, Bcl-2-associated X protein; ERK, extracellular regulated protein kinases; P38, p38 mitogen-activated protein kinase; JNK, c-Jun N-terminal kinase; TFR1, transferrin receptor protein 1; FTH1, ferritin Heavy Chain 1; SLC7A11, solute carrier family 7 member 11; GPX4, glutathione peroxidase 4; β-actin, beta-actin.
